# Modified inflammation-based score as an independent malignant predictor in patients with pulmonary focal ground-glass opacity: a propensity score matching analysis

**DOI:** 10.1038/srep19105

**Published:** 2016-01-11

**Authors:** Long Jiang, Shanshan Jiang, Yongbin Lin, Han Yang, Zerui Zhao, Zehua Xie, Yaobin Lin, Hao Long

**Affiliations:** 1 Sun Yat-sen University Cancer Center, State Key Laboratory of Oncology in South China, Collaborative Innovation Center for Cancer Medicine, Guangzhou 510060, China; 2 Lung Cancer Institute of Sun Yat-sen University, Guangzhou 510060, China; 3 Department of Thoracic Oncology, Sun Yat-sen University Cancer Center, Guangzhou 510060, China; 4 University of California, San Francisco, San Francisco, USA

## Abstract

Pulmonary focal Ground-glass Opacities (fGGOs) would frequently be identified after widely implementation of low-dose computed tomography (LDCT) screening. Because of the high false-positive rate of LDCT, antibiotics should be regarded as advocates in clinical management for detected fGGOs. Retrospectively review consecutive patients with fGGOs between August 2006 and August 2012. Then, relative Glasgow prognostic score (GPS) were constructed in three different systems, traditional GPS system (tGPS), modified GPS system 1 (m1GPS), and modified GPS system 2 (m2GPS). Moreover, propensity score matching (PSM) was employed in balancing baseline covariates. After PSM, patients were matched and included in benign and malignant groups as 1:1 ratio. All reported parameters were balanced in both groups and no statistical differences could be detected. Finally, m1GPS exhibited remarkable different distribution between benign and malignant fGGOs. In detail, m1GPS 1 was more frequently observed in benign fGGOs nodules, while m1GPS 2 in malignant fGGOs nodules. Modified inflammation-based score was identified as an independent predictor of malignancies in patients with pulmonary fGGOs. Patients with m1GPS 1 were more likely to be benign fGGOs, while victims with m1GPS 2 more likely to be malignant.

Pulmonary focal Ground-glass Opacities (fGGOs), defined as “Hazy increased attenuation of lung, but with preservation of bronchial and vascular margins; caused by partial filling of air spaces, interstitial thickening, partial collapse of alveoli, normal expiration, or increased capillary blood volume”^1^, were commonly recognized as nonspecific lung computed tomography (CT) findings^2^. After success of the National Lung Screening Trial^3^, more widely implementation of low-dose computed tomography (LDCT) screening would identify even more nodules^4^, although approximately 150,000 new lung nodules has been detected annually in the United States^5^.

However, the false-positive rate of LDCT was extremely high^6^; and only 1% to 12% detected fGGOs were malignant^7^. Despite the fact that a wide range existed in the differential diagnosis of lung fGGOs^8^, evidences demonstrated the most common cause of lung fGGOs would be infection^9^, especially in developing countries and areas^10^. As a result, antibiotics should be regarded as advocates in clinical management for detected fGGOs to obviate unnecessary follow-up testing, radiation exposure, anxiety, depression, and substantial financial costs^11^. Nevertheless, selecting candidates for antibiotic use remained controversial^12^, which resulted in concerns in the likelihood of meaningful infections among detected nodules and the potential for antibiotics resistance^13^.

In order to better and easier evaluating inflammation status, elevated systemic C-reactive protein (CRP), as a typical index and a sensitive measure of the systemic inflammatory response^14^, and hypoalbuminemia, an indicator of malnutrition^15^, has been combined to construct an inflammation-based score system, named as Glasgow prognostic score (GPS)^16^. Aiming to improve the predictive effect of the GPS system, modified versions of the GPS system were developed, either adjusted cut-off values of both serum CRP and albumin levels^17^, or omitted hypoalbuminaemia alone as a negative prognostic indicator^18^.

In observational non-randomized studies, the baseline characteristics between the compared groups would be statistically different^19^. Specifically, potential confounding factors that might affect the outcomes of benign and malignant fGGOs would be statistically different, which would result in inaccurate assessment of inflammation-based score in patients with pulmonary fGGOs. To minimizing selection bias in non-randomized cohorts, propensity score matching (PSM) has been proposed as a statistical tool since 1983^20^. The constructed score, describing the condition of unbalanced baseline covariates for participants in either experimental or control group^21^, could be used for matching in order to control the confounding between different groups^22^.

Given the fact of increasing detection of lung fGGOs and a paucity of evidence on clinical antibiotics utilities^23^, this preliminary study was designed with the aim of identifying an effective predictor of antibiotics use in treatment after lung fGGOs detection. This subset of victims should be recommended for antibiotics application because of the potential benefits.

## Result

### Clinical outcomes

128 patients with pulmonary fGGOs nodules were eligible for the final analysis. In this group of 128 patients, the mean age was 55.4 years. Additionally, malignant fGGOs were pathologically diagnosed as adenocarcinoma in 26patients, squamous cell carcinoma in 10patients, carcinoma *in situ* in 29 patients, and lymphoepithelioma in 12 patients. Accordingly, benign fGGOs were pathologically diagnosed as tuberculoma in 14 patients, pneumonia in 31 patients, and hamartomastage in 6 patients. Malignant fGGOs nodules were statistically correlated with presence of symptoms (p = 0.007), dominant nodule(s) with part-solid component (p < 0.001), and spiculation (p = 0.017), present of history of lung cancer (p = 0.015) and history of other cancers (p = 0.001), as well as larger lesions (p = 0.001). These imbalance parameters were proved to be risk factors of malignant fGGOs in previous studies and guidelines^2^,^11^,^24^. Other parameters included in guidelines were all reviewed and reported in these 128 patients, although no significantly differences were observed (Tables 1 and 2).

**Figure 1:**
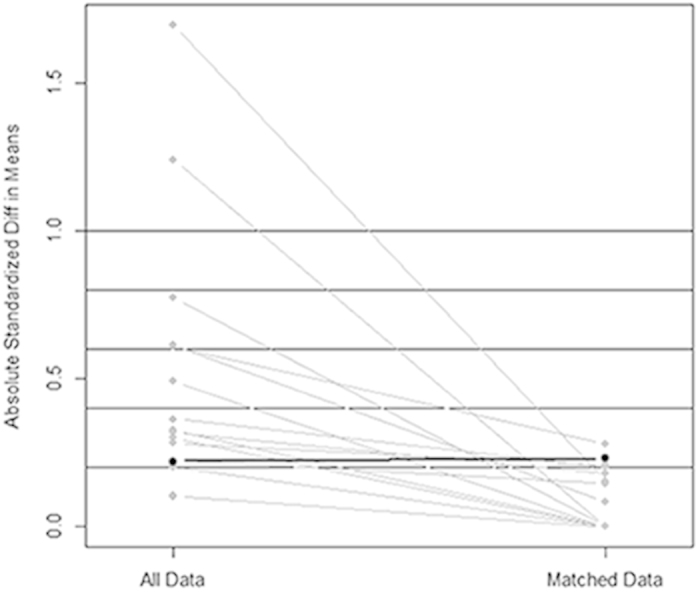
Parallel line plot of the standardized difference in means before and after PSM in patients with benign and malignant fGGOs. As the standardized difference in means was reduced, covariate balance was improved in the matched samples.

**Figure 2:**
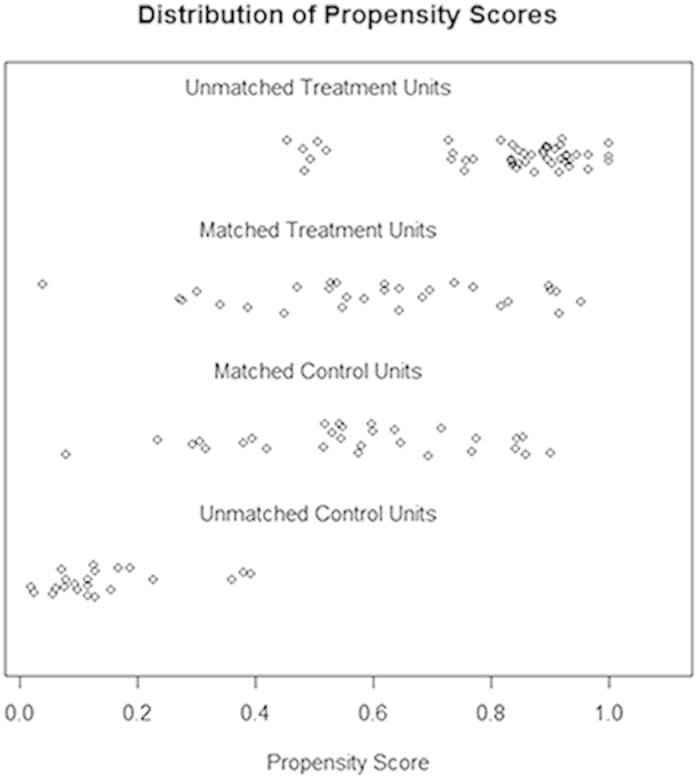
Dot plot of the propensity scores of patients with benign and malignant fGGOs showing individual units in the dataset and whether they were matched or discarded. Treatment units: patients with malignant fGGOs; Control units: patients with benign fGGOs.

All 128 patients (51 benign fGGOs and 77 malignant fGGOs) were eligible for PSM under one-to-one nearest neighbor matching algorithm at a caliper of 0.2. The calculated PS, constructed for the entire 128 cases, ranged from 0.03 to 1.0 and had a median of 0.67. Before matching, the mean propensity score was 0.39 for patients with benign fGGOs (n = 51) and 0.74 for patients with malignant fGGOs (n = 77) (P = 0.003). After PSM under one-to-one nearest neighbor matching algorithm at a caliper of 0.2, 82 patients (41 benign fGGOs and 41 malignant fGGOs) were matched and included in benign and malignant groups. The mean propensity score was 0.46 for patients with benign fGGOs (n = 41) and 0.70 for patients with malignant fGGOs (n = 41) (P = 0.805). The standardized difference in means and distribution of propensity scores consistently illustrated improvement of covariate balance after PSM (Figs 1 and 2). In this group of 82 patients, the mean age was 53.5 years. All reported parameters were balanced in both groups and no statistical differences could be detected, including symptoms (p = 0.372), dominant nodule(s) with part-solid component (p = 1.000), spiculation (p = 0.262), history of lung cancer (p = 0.312), history of other cancers (p = 0.198), and tumor size (p = 0.160) (Tables 1 and 2). Additionally, histopathological analyses showed no significant difference before and after PSM (p = 0.152), which illustrated consistent outcomes of benign and malignant fGGOs before and after the balancing procedure of PSM, thus confirming the reliability of PSM in balancing baseline demographic characteristics (Table S1).

### PSM analysis

Before PSM, the distribution of tGPS score was tGPS 0 in 22 (43.1%) patients, tGPS 1 in 21 (41.2%) patients, and tGPS 2 in 8 (15.7%) of benign fGGOs; and tGPS 0 in 27 (35.1%) patients, tGPS 1 in 33 (42.8%) patients, and tGPS 2 in 17 (22.1%) of malignant fGGOs, accordingly (Table 3). Additionally, the distribution of m1 GPS score was m1 GPS 0 in 26 (51.0%) patients, m1 GPS 1 in 17 (33.3%) patients, and m1 GPS 2 in 8 (15.7%) of benign fGGOs; and m1 GPS 0 in 30 (39.0%) patients, m1 GPS 1 in 30 (39.0%) patients, and m1 GPS 2 in 17 (22.0%) of malignant fGGOs, accordingly (Table 4). Furthermore, the distribution of m2 GPS score was m2 GPS 0 in 17 (33.3%) patients, m2 GPS 1 in 18 (35.3%) patients, and m2 GPS 2 in 16 (31.4%) of benign fGGOs; and m2 GPS 0 in 20 (26.0%) patients, m2 GPS 1 in 17 (22.1%) patients, and m2 GPS 2 in 40 (51.9%) of malignant fGGOs, accordingly (Table 5). Consequently, no statistical differences could be observed between benign and malignant fGGOs nodules among these 128 patients in aspects of tGPS (p = 0.553), m1GPS (p = 0.383) and m2GPS (p = 0.064) (Tables 3, 4, 5).

After PSM, the distribution of tGPS score was tGPS 0 in 17 (41.5%) patients, tGPS 1 in 17 (41.5%) patients, and tGPS 2 in 7 (17.0%) of benign fGGOs; and tGPS 0 in 15 (36.6%) patients, tGPS 1 in 17 (41.5%) patients, and tGPS 2 in 9 (21.9%) of malignant fGGOs, accordingly (Table 3). Additionally, the distribution of m1 GPS score was m1 GPS 0 in 13 (31.7%) patients, m1 GPS 1 in 22 (53.7%) patients, and m1 GPS 2 in 6 (14.6%) of benign fGGOs; and m1 GPS 0 in 10 (24.4%) patients, m1 GPS 1 in 3 (7.3%) patients, and m1 GPS 2 in 28 (68.3%) of malignant fGGOs, accordingly (Table 4). Furthermore, the distribution of m2 GPS score was m2 GPS 0 in 13 (31.7%) patients, m2 GPS 1 in 15 (36.6%) patients, and m2 GPS 2 in 13 (31.7%) of benign fGGOs; and m2 GPS 0 in 10 (24.4%) patients, m2 GPS 1 in 10 (24.4%) patients, and m2 GPS 2 in 21 (51.2%) of malignant fGGOs, accordingly (Table 5). Interestingly, although significant differences still could not be observed between benign and malignant fGGOs nodules in aspects of tGPS (p = 0.829) and m2GPS (p = 0.195) (Tables 3 and 5), m1GPS exhibited remarkable different distribution between benign and malignant fGGOs (p < 0.001) (Table 4). In detail, m1GPS 1 was more frequently observed in benign fGGOs nodules, while m1GPS 2 in malignant fGGOs nodules. This interesting result was caused by the different definition of different GPS systems. Elevated CRP level, representing an inflammation cause of the host, would be more likely represent an inflammation cause for fGGOs, instead of hypoalbuminemia, representing malnutrition of the host. Furthermore, the suitable cut-off values should be 10 mg/L for elevated CRP level and 35 g/L for hypoalbuminemia.

## Discussion

The current preliminary study, after evaluating and comparing different inflammation-based score systems, identified m1GPS as an effective predictor of antibiotics use in treatment after lung fGGOs detection. A subset of victims should be chosen for antibiotics in application because of the potential benefits.

After widely application of LDCT in lung screening, pulmonary fGGOs would be frequently identified^24^. fGGOs were considered to be a great challenge for biopsy due to their small size or unnecessary for immediate aggressive diagnostic procedures but only referred for follow-up with series of CT scans because of low risk for malignancy^25^. Nonetheless, such a strategy would be expected to result in significant anxiety, radiation exposure, and additional cost^26^. By contrast, a safe, simple and inexpensive option, such as antibiotics prescription, should be reckoned in fGGOs management^23^. Although antibiotics prescription was supported by their effectiveness against plenty of inflammatory disorders causing fGGOs, indications and exact utilities of antibiotics prescription in fGGOs remained unclear^23^,^27^. Even if clinicians suggested some clinical and radiographic characteristics and an improving trend with antibiotic use, no statistical associations between patients’ characteristics and antibiotics use could be discovered in previous studies^23^,^28^. In the present study, potential risk factors and inflammation-based score systems were analyzed though PSM method to identify a subset of candidates of antibiotics prescription with probable benefits.

In real world, treatment selection was usually influenced by a series of baseline characteristics^29^. For this reason, baseline characteristics should be taken into consideration when accessing therapy regimens^30^. PSM, designed for reducing or eliminating differences among baseline characteristics, was attracting increasing interests in medical research^31^,^32^. Before PSM, some demographic characteristics were imbalance, which might affect the outcomes of benign and malignant fGGOs , thus confounding the real role of inflammation-based score in patients with pulmonary fGGOs. After PSM, both groups illustrated similar demographic characteristics with no significant differences, which suggested that PSM effectively minimized imbalance among covariates.

Existed investigations have proved the inflammation-based prognostic score, GPS, as predictor for coexistence of systemic inflammation and malnutrition of the host^33^. GPS could be considered routinely applied globally depended on its plain, minimally invasive, and cost-effect measurement^34^. Furthermore, considerable attention was poured into improving the predict effect of GPS^18^,^35^. Some investigators modified the cut lines of abnormal serum albumin and CRP level at 38 g/L and 5 mg/L, respectively^35^. Additionally, other studies recommended another GPS modification as assigning normal CRP but hypoalbuminemia to GPS 0 group^18^. All three GPS systems were evaluated in identifying the antibiotics beneficial. Finally, only m1GPS, allocated hypoalbuminemia alone to GPS 0, was proved as an effective predictor of malignancies in patients with pulmonary fGGOs. This could be explained as that systemic inflammation would be more likely represent an inflammation cause for fGGOs, instead of malnutrition of the host. Moreover, patients with m1GPS 1 were more likely to be benign fGGOs and with m1GPS 2 malignant, while no significant different between benign and malignant fGGOs in m1GPS 0 group. A possible interpretation might be inflammation caused fGGOs would be resulting in systemic malnutrition. Thus, if both systemic inflammation and malnutrition coexisted, the fGGOs would be a higher probability of malignancies.

Although other covariates involving dynamic change during follow-up were also included in the guidelines^2^,^36^, the present study focus on the clinical management of first detected fGGOs. Due to this reason, only covariates associated with the first detection were included. Besides, as a retrospective study, clinical and survival comparison might be dependent on selection bias due to its retrospective nature. Even if PSM could significantly overcome this limitation, future prospective multi-institutional large-scale studies were still a need in validating the findings.

In conclusion, modified inflammation-based score was identified as an independent predictor of malignancies in patients with pulmonary fGGOs. Patients with m1GPS 1 were more likely to be benign fGGOs, while victims with m1GPS 2 more likely to be malignant. This pilot conclusion should be evaluated in future prospective studies involving antibiotics prescription to further clarify the clinical role of the GPS system in patients with fGGOs.

## Method

Study protocol was approved by the institutional review boards of Sun Yat-Sen University Cancer Center (SYSUCC). Written informed consent was obtained from each patient: including signed consent for tissue analysis as well as consent to be recorded for potential medical research at the time of patients’ admission. All experiments were performed in accordance with relevant guidelines and regulations.

### Patients

Chart review was performed on consecutive patients who had undergone CT scans at SYSUCC between August 2006 and August 2012. Both clinical and pathological data were collected and reviewed. Only patients with pulmonary fGGO lesions were recruited if definitive diagnosis of malignant or benign were recorded concurrently at SYSUCC. Moreover, patients with pulmonary fGGO lesions were excluded from analysis if: 1) lacking qualifying CT scans, 2) with history of primary lung cancer or any other malignancies and systemic treatment (i.e. chemotherapy) were not finished at the time of detecting fGGO lung nodules, 3) diagnosis of any other malignancies during follow-up period. Under these criteria, patients underwent primary lung cancer or any other malignancies, but without ongoing systemic treatment when fGGOs detection, were included in final analysis.

The fGGOs was judged as classic definition. 50% GGO area was set as the cut-off value in identifying solid lesions or dominant nodule(s) with part-solid component^37^. Furthermore, multiple fGGOs were also included, because of occasional reports of multicentric lung adenocarcinoma^38^. In addition, since relatively large pure fGGOs were pathologically diagnosed as adenocarcinomas, fGGOs size was not considered as an exclusion criterion^39^.

Malignant fGGOs were defined as malignant diagnosis by pathologic examination of tissue obtained via surgery or biopsy. Accordingly, benign fGGOs were defined as either pathologic examination of tissue obtained via surgery or biopsy or fGGOs resolving during follow-up. However, in the latter situation, the exact classification was recommended as non-malignant lesion because no pathological diagnoses available. All pathological data were reviewed and confirmed by two independent pathologists based on WHO classification of Lung Cancer^40^.

CT scans were performed by a Toshiba Aquilion 64 CT scanner (Toshiba American Medical Systems Inc, Tustin, CA) during one breath-hold with 5-mm reconstruction and 2-mm slice collimation. Both lung (width, 1,500 HU; level, –700 HU) and mediastinal (width, 400 HU; level, 20 HU) window images were obtained and reviewed. All fGGO nodules characteristics were examined by thin-section chest CT scans (section thickness < 2.5 mm). The size of fGGO was measured as maximal diameter at lung window^41^. The fGGO lesions were classified as pure GGO and dominant nodule(s) with part-solid component based on the tumor shadow disappearance rate (TDR): dominant nodule(s) with part-solid component (0 < TDR < 1), and pure GGO (TDR = 0)^42^. All radiographic images were reviewed and confirmed by the same thoracic surgeon and consultant radiologist. The final decision for each radiology finding was made by consensus between them.

### GPS system

In GPS evaluation, laboratory examinations including CRP and albumin were performed within 24 hours before or after CT scans as routine clinical practice in SYSUCC. Serum CRP and albumin levels were examined by the Hitachi Auto Analyzer (Hitachi 7600, Hitachi, Tokyo, Japan). The inter- and intra-assay variability of CRP and albumin concentrations were less than 5% as established by routine quality control procedures.

Relative GPS systems were constructed as previous reports^35^. In traditional GPS system, victims with both hypoalbuminemia (<35 g/L) and elevated CRP level (>10 mg/L) were allocated into tGPS 2 group. And, patients with neither of these two abnormalities were allocated into tGPS 0 group. Then, remaining patients with only one biochemical abnormalities were allocated into tGPS 1 group. Differently, in modified GPS system 1, patients with hypoalbuminaemia (<35 g/L) alone were classified into m1GPS 0 group, while other criteria for m1GPS score is the same with tGPS system. In detail, m1GPS 1 was defined as patients with elevated CRP level (>10 mg/L) alone, while m1GPS 2 as patients with both hypoalbuminemia (<35 g/L) and elevated CRP level (>10 mg/L). Additionally, in modified GPS system 2, the cut-off values were changed as 5 mg/L for elevated CRP level and 38 g/L for hypoalbuminemia. Other score assigning criteria for m2GPS system is the same with tGPS system (Table 6).

### Statistical analysis

Categorical data were presented as numbers and percentages and continuous data as median and range unless otherwise stated. The Pearson χ^2^ test and McNemar’s test were used for categorical data, and an independent sample t-test or the Mann–Whitney U test were used for numerical data. P < 0.05 was considered to be significant in all statistical analyses.

Variables with statistically significant differences between groups might have impact on the postoperative outcomes. The PSM, aiming to minimize the influence of selection bias and potential confounding variables between benign and malignant fGGOs, was generated using all reported covariates with one-to-one nearest neighbor matching algorithm at a caliper of 0.2. The included characteristics as covariates were age, smoking history (measured by pack-yr), time since smoking cessation, sex, symptoms (including cough, dyspnea, sputum production, wheezing, night sweats, fever and weight loss), history of other lung diseases (including chronic obstructive pulmonary disease and pulmonary fibrosis), history of lung cancer, history of other cancers, family history of lung cancer, fGGOs size, GGO numbers, GGO type, cavitation, spiculation, and calcification. The standardized difference in means and distribution of propensity scores were used in assessing the improvement of covariate balance after PSM. The propensity score was calculated by multiplying the coefficient for each variable in the model. The initial unmatched and matched samples were assessed by calculating standardized differences. A standardized difference of less than the absolute value of 0.2 was taken to indicate negligible difference in the mean or prevalence of a covariate between the compared groups^43^. All the above procedures, inclusion calculation and matching, could be conducted by IBM SPSS Statistics for Windows and SPSS PS Matching plug-in.

Data management and statistical analyses were performed using IBM SPSS Statistics (IBM SPSS Statistics for Windows, Version 22.0. IBM Corp., Armonk, NY) for Windows (SPSS Inc, Chicago, IL) and SPSS PS Matching plug-in (Propensity score matching in SPSS, psmatching3.03, Felix Thoemmes, Cornell University/University of Tübingen).

## Additional Information

**How to cite this article**: Jiang, L. *et al*. Modified inflammation-based score as an independent malignant predictor in patients with pulmonary focal ground-glass opacity: a propensity score matching analysis. *Sci. Rep*. **6**, 19105; doi: 10.1038/srep19105 (2016).

## Supplementary Material

Supplementary Information

## Figures and Tables

**Table 1:** Clinical characteristics of benign and malignant fGGOs before and after PSM.

Characteristic	Before PSM					After PSM				
	All (n = 128)		Benign (n = 51)	Malignant (n = 77)	P	All (n = 82)		Benign (n = 41)	Malignant (n = 41)	P
Age, yrs	56.6† (range: 18–79)		53.8† (range: 18–77)	58.7† (range: 33–79)	0.078	54.4† (range: 18–79)		55† (range: 18–77)	54† (range: 33–79)	0.817
Smoking history, pack-yr	7.8^†^ (range: 0–60)		9.7† (range: 0–60)	6.6† (range: 0–60)	0.587	5.9† (range: 0–60)		9.3† (range: 0–60)	2.5† (range: 0–60)	0.173
Time since smoking cessation, yr	0.8^†^ (range: 0–20)		1.2† (range: 0–20)	0.6† (range: 0–20)	0.204	1.1† (range: 0–20)		1.3† (range: 0–20)	0.9† (range: 0–20)	0.340
Sex(%)					0.149					0.659
Male	62	48.4%	29	33		41	50.0%	22	19	
Female	66	51.6%	22	44		41	50.0%	19	22	
Symptom					0.007					0.372
Absent	69	53.9%	35	34		47	57.3%	26	21	
Present	59	46.1%	16	43		35	42.7%	15	20	
History of other lung diseases					0.702					1.000
Absent	121	94.5%	49	72		80	97.6%	40	40	
Present	7	5.5%	2	5		2	2.4%	1	1	
History of lung cancer					0.015					0.312
Absent	112	87.5%	40	72		72	87.8%	34	38	
Present	16	12.5%	11	5		10	12.2%	7	3	
History of other cancers					0.001					0.198
Absent	98	76.6%	31	67		62	75.6%	28	34	
Present	30	23.4%	20	10		20	24.4%	13	7	
Family history of lung cancer					0.682					0.999
Absent	122	95.3%	48	74		77	93.9%	38	39	
Present	6	4.7%	3	3		5	6.1%	3	2	

PSM: propensity score matching.

^†^Values are given as the median fGGOs.

**Table 2:** Radiological characteristics of benign and malignant fGGOs before and after PSM.

Characteristic	Before PSM					After PSM				
	All (n = 128)		Benign (n = 51)	Malignant (n = 77)	P	All (n = 82)		Benign (n = 41)	Malignant (n = 41)	P
Size of fGGOs, mm	21.5† (range: 5–45)		16.0† (range: 5–45)	26.0† (range: 8–45)	0.001	20.0† (range: 5–45)		16.0† (range: 5–45)	22.0† (range: 8–45)	0.160
GGO numbers					0.194					0.547
Solitary	110	85.9%	41	69		69	84.1%	33	36	
Multiple	18	14.1%	10	8		13	15.9%	8	5	
GGO type					<0.001					1.000
Pure GGO without a dominant lesion(s)	43	33.6%	31	12		42	51.2%	21	21	
Dominant nodule(s) with part-solid component	85	66.4%	20	65		40	48.8%	20	20	
Cavitation					0.062					0.116
Present	17	13.3%	3	14		12	14.6%	3	9	
Absent	111	86.7%	48	63		70	85.4%	38	32	
Spiculation					0.017					0.262
Present	52	40.6%	14	38		34	41.5%	14	20	
Absent	76	59.4%	37	39		48	58.5%	27	21	
Calcification					0.275					0.241
Present	3	2.3%	0	3		3	3.7%	0	3	
Absent	125	97.7%	51	74		79	96.3%	41	38	

fGGOs: focal Ground-glass Opacity.

PSM: propensity score matching.

^†^Values are given as the median.

**Table 3:** Distribution of traditional GPS in patients with benign and malignant fGGOs before and after PSM.

	Before PSM (n = 128)				After PSM (n = 82)			
	tGPS 0, n (%)	tGPS 1, n (%)	tGPS 2, n (%)	Total, n	tGPS 0, n (%)	tGPS 1, n (%)	tGPS 2, n (%)	Total, n
Benign, n (%)	22 (43.1%)	21 (41.2%)	8 (15.7%)	51	17 (41.5%)	17 (41.5%)	7 (17.0%)	41
Malignant, n (%)	27 (35.1%)	33 (42.8%)	17 (22.1%)	77	15 (36.6%)	17 (41.5%)	9 (21.9%)	41

fGGOs: focal Ground-glass Opacity.

PSM: propensity score matching.

tGPS: traditional Glasgow prognostic score system.

**Table 4:** Distribution of modified GPS 1 in patients with benign and malignant fGGOs before and after PSM.

	Before PSM (n = 128)				After PSM (n = 82)			
	m1GPS 0, n (%)	m1GPS 1, n (%)	m1GPS 2, n (%)	Total, n	m1GPS 0, n (%)	m1GPS 1, n (%)	m1GPS 2, n (%)	Total, n
Benign, n (%)	26 (51.0%)	17 (33.3%)	8 (15.7%)	51	13 (31.7%)	22 (53.7%)	6 (14.6%)	41
Malignant, n (%)	30 (39.0%)	30 (39.0%)	17 (22.0%)	77	10 (24.4%)	3 (7.3%)	28 (68.3%)	41

fGGOs: focal Ground-glass Opacity.

PSM: propensity score matching.

m1GPS: modified Glasgow prognostic score system 1.

**Table 5:** Distribution of modified GPS 2 in patients with benign and malignant fGGOs before and after PSM.

	Before PSM (n = 128)				After PSM (n = 82)			
	m2GPS 0, n (%)	m2GPS 1, n (%)	m2GPS 2, n (%)	Total, n	m2GPS 0, n (%)	m2GPS 1, n (%)	m2GPS 2, n (%)	Total, n
Benign, n (%)	17 (33.3%)	18 (35.3%)	16 (31.4%)	51	13 (31.7%)	15 (36.6%)	13 (31.7%)	41
Malignant, n (%)	20 (26.0%)	17 (22.1%)	40 (51.9%)	77	10 (24.4%)	10 (24.4%)	21 (51.2%)	41

fGGOs: focal Ground-glass Opacity.

PSM: propensity score matching.

m2GPS: modified Glasgow prognostic score system 2.

**Table 6:** Allocation of different inflammation-based score systems.

Biochemical abnormalities	Score
Neither hypoalbuminemia (<35 g/L) nor elevated CRP level (>10 mg/L)	tGPS 0
Either hypoalbuminemia (<35 g/L) or elevated CRP level (>10 mg/L)	tGPS 1
Both hypoalbuminemia (<35 g/L) and elevated CRP level (>10 mg/L)	tGPS 2
Without elevated CRP level (>10 mg/L)	m1 GPS 0
Elevated CRP level (>10 mg/L) but without hypoalbuminemia (<35 g/L)	m1 GPS 1
Both hypoalbuminemia (<35 g/L) and elevated CRP level (>10 mg/L)	m1 GPS 2
Neither hypoalbuminemia (<38 g/L) nor elevated CRP level (>5 mg/L)	m2 GPS 0
Either hypoalbuminemia (<38 g/L) or elevated CRP level (>5 mg/L)	m2 GPS 1
Both hypoalbuminemia (<38 g/L) and elevated CRP level (>5 mg/L)	m2 GPS 2

tGPS: traditional Glasgow prognostic score system.

m1GPS: modified Glasgow prognostic score system 1.

m2GPS: modified Glasgow prognostic score system 2.
