# Supplementary material for: Modified inflammation-based score as an independent malignant predictor in patients with pulmonary focal ground-glass opacity: a propensity score matching analysis
Source: Sci Rep. 2016 Jan 11;6:19105. doi: 10.1038/srep19105 (PMC4707538; doi:10.1038/srep19105)
Supplement: Supplementary Information [file srep19105-s1.pdf]

Title

Modified inflammation-based score as an independent malignant predictor in patients with pulmonary focal ground-glass opacity: a propensity score matching analysis

Authors

Long Jiang*[1,2,3,4] , Shanshan Jiang*[1] , Yongbin Lin[1,2,3] , Han Yang[1,2,3] , Zerui Zhao[1,2,3] , Zehua Xie[1,2,3] , Yaobin Lin[1,2,3] , and Hao Long[1,2,3]

*These authors contributed equally to this article.

Supplemental Table S1

Table S1 Histopathological characteristic before and after PSM

Characteristic	Before PSM (n=128)		After PSM (n=82)		P
Histopathological results					0.152
Adenocarcinoma	26	20.3%	12	14.6%	
Squamous cell carcinoma	10	7.8%	5	6.2%	
Carcinoma in situ	29	22.7%	17	20.7%	
Lymphoepithelioma	12	9.4%	7	8.5%	
Tuberculoma	14	10.9%	11	13.4%	
Pneumonia	31	24.2%	27	32.9%	
Hamartoma	6	4.7%	3	3.7%	

PSM: propensity score matching
